# Application of Genetically Encoded Photoconvertible Protein SAASoti for the Study of Enzyme Activity in a Single Live Cell by Fluorescence Correlation Microscopy

**DOI:** 10.3390/ma15144962

**Published:** 2022-07-16

**Authors:** Ilya D. Solovyev, Liliya G. Maloshenok, Alexander P. Savitsky

**Affiliations:** 1 A.N. Bach Institute of Biochemistry, Research Center of Biotechnology of the Russian Academy of Sciences, 33, bld. 2 Leninsky Ave., 119071 Moscow, Russia; i.solovyev@fbras.ru; 2 N.I. Vavilov Institute of General Genetics Russian Academy of Sciences, Gubkina Str. 3, 119333 Moscow, Russia; maloshenoklg@gmail.com

**Keywords:** FCS, FLIM, FRET sensor, photoconvertible FP, caspase, single cell

## Abstract

Fluorescent Correlation Spectroscopy (FCS) allows us to determine interactions of labeled proteins or changes in the oligomeric state. The FCS method needs a low amount of fluorescent dye, near nanomolar concentrations. To control the amount of fluorescent dye, we used new photoconvertible FP SAASoti. This work is devoted to the proof of principle of using photoconvertible proteins to measure caspase enzymatic activity in a single live cell. The advantage of this approach is that partial photoconversion of the FP makes FCS measurements possible when studying enzymatic reactions. To investigate the process, in vivo we used HeLa cell line expressing the engineered FRET sensor, SAASoti-23-KFP. This FRET sensor has a cleavable (DEVD) sequence in the linker between two FPs for the detection of one of the key enzymes of apoptosis, caspase-3. Caspase-3 activity was detected by registering the increase in the fluorescent lifetimes of the sensor, whereas the diffusion coefficient of SAASoti decreased. This can be explained by an increase in the total cell viscosity during apoptosis. We can suppose that in the moment of detectible caspase-3 activity, cell structure already has crucial changes in viscosity.

## 1. Introduction

Genetically encoded colored and fluorescent proteins are widely used in modern research in cell biology [1]. The use of fluorescent proteins make it possible to study a variety of molecular processes in living cells [2,3,4]. However, the application of the methods of fluorescence correlation spectroscopy is rather limited. Fluorescence Correlation Spectroscopy (FCS) is based on the fluctuation of a fluorescence signal in a small volume. At low fluorophore concentrations, the focal volume contains one to several fluorophore molecules. By diffusing through the focal volume, the fluorophore generates fluctuations in the fluorescence signal. Thus, it is possible to determine the oligomeric state of fluorescent proteins in highly diluted solutions, which was first demonstrated for GFP, since it has a monomeric form at low concentrations [5]. This method only detects fluorescent molecules, so it can be used in complex systems and in cells [6,7]; however, its use is limited to the concentration range of measurements. The method of fluorescence correlation spectroscopy requires the use of a fluorophore concentration in the range of 10^−8^–10^−9^ M, and in studies in single living cells, the overexpression of fluorescent proteins will be observed.

With commonly used constitutive promoters, controlling the fluorescent proteins expression in a cell is a difficult task. Most often, protein genes for expression in mammalian cells are placed under constitutive promoters (CMV, etc.); as a result, the concentration continuously increases, and from a certain point in time, begins to exceed the concentration at which it is possible to use the FCS method. Special costly promoters have been used for low expression levels [8]. This method of obtaining low expression levels partially solves the problem of concentration for a certain range of tasks not bound by a rigid time interval when the measurement should be taken, since it does not take into account the gradual accumulation of fluorescent protein, which creates a sufficiently long lag period to reach the required concentration level, and in long-term experiments in the absence of protein catabolism, it still leads to an excess of the required concentrations. At the same time, the method is rather labor-intensive and requires a significant amount of time to create the necessary structures. The level of expression is more difficult to manage than to optimize the light dose for photoconversion. Therefore, for the necessary control over the concentration of the fluorophore, it was proposed to use a photoconvertible fluorescent protein. In principle, for FCS, any photoconvertible fluorescent protein can be used. For the first time, photoactivatable proteins for the classical FCS method were applied in 2017 [9], and photoconvertible proteins for the correlation method in a plane laser beam were used in a similar scheme with Dendra2 [10]. We have shown this on the example of the protein SAASOti.

Photoconversion is an irreversible photochemical reaction of the transition of a fluorescent protein from green to red under the action of irradiating light λ = 400 nm. This process is typical for fluorescent proteins that have a conservative triad of amino acid residues—HYG—in the chromophore. For the first time, the phenomenon of photoconversion was observed for the fluorescent protein Kaede [11]. The absorption maximum of the green form λ = 400 nm corresponds to the protonated form of the chromophore. Most often, only the protonated form of the chromophore undergoes this phototransformation. In this case, a beta-elimination reaction occurs, the cleavage of the bond between Cα and Nα atoms of the chromophore-forming conservative amino acid residue of histidine and the formation of a double bond between Cα and Cβ of the same residue, which is conjugated with the π-system of the chromophore. As a result, there is a spectral shift in the excitation and emission of the chromophore to the red region [12]. However, the reaction mechanism still causes some controversy: the transfer of a proton in an excited state and one-electron transfer have not been confirmed, and Ref. [13] found that amino acid residues H193, E211, and Q38 of the chromophore microenvironment play an important role in this process. When obtaining a photoconvertible derivative of the fluorescent protein GFP, it was found that in addition to the histidine residue included in the chromophore, the residues of the nearest environment of the chromophore (Q38, H193, E211), which ensure its mobility, also play an important role.

The purpose of this work is to demonstrate the possibility of using the FCS method to study enzymatic activity in vivo in this case for one of the enzymes that plays a key role in programmed cell death, or apoptosis. Caspases are among the most important cell enzymes, as they control cell functioning, including programmed cell death, and caspase-3 is the terminal in the cell death program [14,15]. Measurements at one single point in time do not make sense, since during apoptosis, a significant change in cell morphology occurs. Earlier, our laboratory developed a FRET sensor for caspase-3 based on TagRFP fluorescent protein and non-fluorescent chromoprotein KFP [16], which are interconnected by a linker of 23 amino acid residues containing in the middle a DEVD sequence for cleavage by caspase-3 [17,18,19]. The fluorescence lifetime of free TagRFP is 2.4 ns, and upon the implementation of resonant energy transfer with KFP, which is in a dark form and does not fluoresce, the fluorescence lifetime of TagRFP is reduced to 1.8 ns. Inside a cell transfected with such a construct, during apoptosis, caspase-3 is activated and the process of sensor cleavage begins, while an increase in the lifetime and intensity of TagRFP fluorescence is recorded.

The goal of this work was to use the FCS method to register enzymatic activity in one single cell. In order to use the FCS method in addition to FRET, we used the SAASoti fluorescent protein, isolated for the first time from the coral *Stylocoeniella armata* [20]. It was previously shown that upon irradiation with violet light λ = 405 nm, SAASoti irreversibly converted from a green fluorescent form into a red one [20,21]. In cell biology, special versions of fluorescent proteins are sometimes required, and there is no universal solution for all problems or a universal optimization for any type of measurement. To use this protein in certain conditions of cellular compartments in live cells, special versions are being developed; for example, for measurements under oxidizing conditions (for example, EPR, Golgi apparatus), cysteine residues are replaced. The photochemistry of biphotochromic proteins is quite complex, and separate publications are devoted to this [22,23]. For cytoplasm and nuclear localization of SAASoti, no special mutants are required (see Materials and Methods). The SAASoti-23-KFP genetic construct was obtained by replacing the TagRFP gene in the previous TagRFP-23-KFP construct in the pcDNA3 eukaryotic vector. KFP is a tetrameric protein, and together with a monomeric donor forms an octameric (according to the number of individual molecules in the sensor) structure, and the elimination of the monomeric donor during sensor hydrolysis is detectable by the change in the diffusion coefficient. The hydrodynamic radius in this case depends on the molecular mass, such as cubic root according to the spherical protein globule approximation. Low fraction amount and double difference at the logarithmic scale of the autocorrelation curve may not be detectable at the early times of cleavage.

## 2. Materials and Methods

**Cell lines.** For experiments with mammalian cells, HeLa cell lines were used. The cells were cultured in DMEM medium (PanEko, Moscow, Russia) containing 5% bovine blood serum in the presence of antibiotics (penicillin 50 U/ml and streptomycin 50 μg/ml). After 2 passages, the cells were transferred onto 35 mm Ibidi plates (Gräfelfing, Germany) with a bottom of 0.15 mm for microscopy, after treatment with a trypsin solution containing 0.5% EDTA (PanEko, Moscow, Russia). At a confluence of more than 50%, transfection was performed with pcDNA3 plasmids containing the free SAASoti and SAASoti-23-KFP genes, using the Torpedo reagent (Ibidi, Gräfelfing, Germany) according to the manufacturer’s protocol. One day after transfection, the appearance of fluorescence was observed. When working with the SAASoti-23-KFP sensor, apoptosis was activated by adding staurosporine with a final concentration of 1 μM in the medium. The observation was carried out 1 h after the addition of staurosporine. 

**Fluorescence correlation spectroscopy (FCS) and time-resolved fluorescence microscopy (FLIM).** The measurements were carried out on an Olympus IX-71 inverted fluorescence microscope (Shinjuku ku, Japan) as part of a PicoQuant MicroTime 200 confocal scanning system (Berlin, Germany). For measurements, we used Olympus objective 100×/1.49 UApo N TIRF with oil immersion. To excite fluorescence in a confocal mode, picosecond pulsed lasers 405 nm, 478 nm, and 532 nm PicoQuant (Berlin, Germany) were used. A Lumencor SpectraX LED light source (Beaverton, OR, USA) was used for wide-field excitation and photochemical transformations. Registration was carried out using 500 LP (green channel), 550 LP (red channel) Chroma (Irvine, CA, USA), 509/22 (green channel), and 580/14 (red channel) Semrock (Rochester, NY, USA) filters. The fluorescent signal was recorded using SPAD detectors from Perkin Elmer (Rodgau, Germany) and MPD (Bolzano, Italy) using a 50 µm pinhole. Data analysis was performed using the PicoQuant SymPhoTime 32 v.5.3.2.2 software package. To systematize the calculated parameters and subsequent data processing, we used Python 3.7 with NumPy and matplotlib libraries. 

The critical parameter for FCS is the shape of the focal volume; therefore, it requires standardization with the known fluorescent dye. However, cells have a complex structure with a changing refractive index, which is why such standardization may be incorrect. Data on some average refractive index from cell to cell are not applicable, since it is obvious that at the single cell resolution, we are measuring in various cellular compartments, in which the refractive index varies from 1.35 to 1.6, which is more than 15% [24]. According to [25], the refractive index has a significant impact on the confocal volume. If the refractive index varied in the range 1.33–1.46, the focal volume changed by 1.4-fold. When working with living cells, the refractive index will change not only from cell to cell, but also within each individual cell as the cell develops through different stages of the cell cycle. Due to the heterogeneous environment in the cell, we cannot talk about the constancy of the focal volume. Only the dynamics of changes in relation to the starting value matter, which is why we analyze and compare only the initial measured parameters—diffusion times.

**Figure 1:**
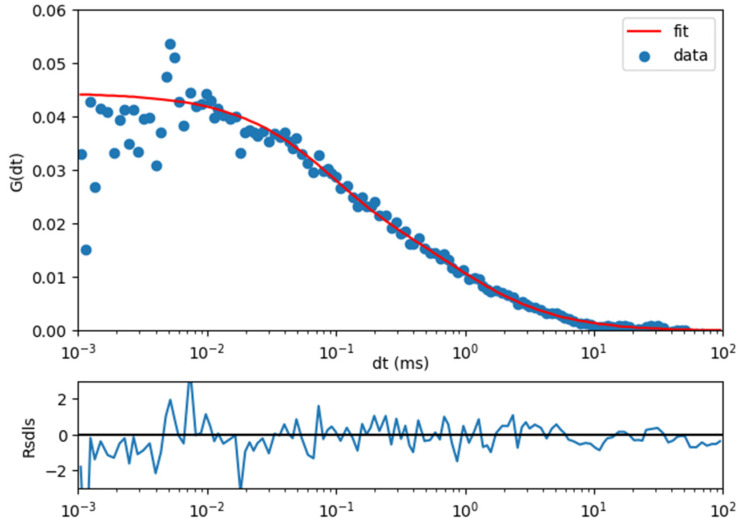
An autocorrelation curve for a triplet model for red SAASoti.

**Figure 2:**
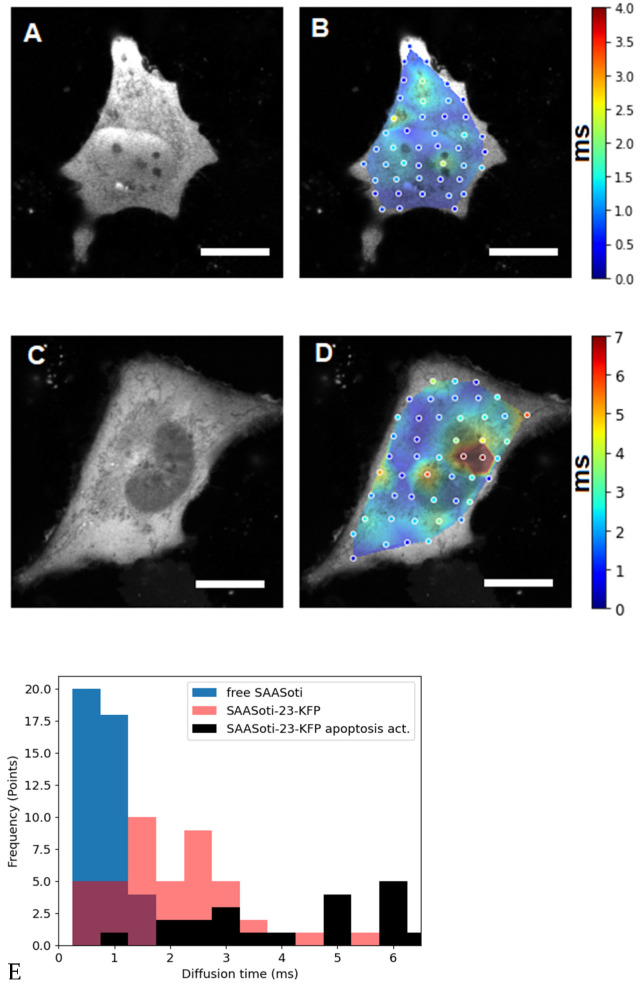
The left panels (**A**,**C**) are fluorescence images of HeLa cells transfected with (**A**) free SAASoti and (**C**) SAASoti-23-KFP in the red channel. The right panels (**B**,**D**) are images of the diffusion time distributions of the SAASoti red form in the cell. The color corresponds to the value of the diffusion time according to the scale to the right of the images in milliseconds. Images are 80 × 80 microns in size. (**E**) Points diagram distribution from (0.5 ms bining) for free SAASOti (**B**), SAASOti-23-KFP (**D**), and SAASOti-23-KFP 2 h after induction of apoptosis by staurosporine (Figure 3D). Scale bar = 20 μm.

The measurement of the green form of SAASOti is impossible in principle, since its concentration is very high. The rate of protein accumulation depends on the type of promoter; in our case, we used a fast promoter, CMV [17,18,19], and it is impossible to observe concentration fluctuations in the focal volume at the single molecule level. Optimization parameters for in vitro measurements of SAASoti are described in [21]. Before FCS measurements in the live cell, the optimal concentration of the red SAASoti form was selected on the cells. Optimization of the signal/background ratio was carried out by changing the duration of the light pulse by 405 nm and is evaluated according to the quality of the correlation curve shown in Figure 1. The focus of the objective was set at a random point in the cell cytoplasm. In the wide-field mode, light flashes of 405 nm with a duration of 1 s and a power of 5% of the LED power were supplied, thus irradiating the entire cell. After that, the fluorescent signal from the point was subjected (5 s exposition time) to correlation analysis, and the height of the correlation curve and “noise” were estimated. When obtaining an optimal curve for analysis (Figure 1), a grid (Figure 2B) of points with a constant step of about 1 μm was installed, evenly distributed over the cell, and the signal was recorded at each point.

**Figure 3:**
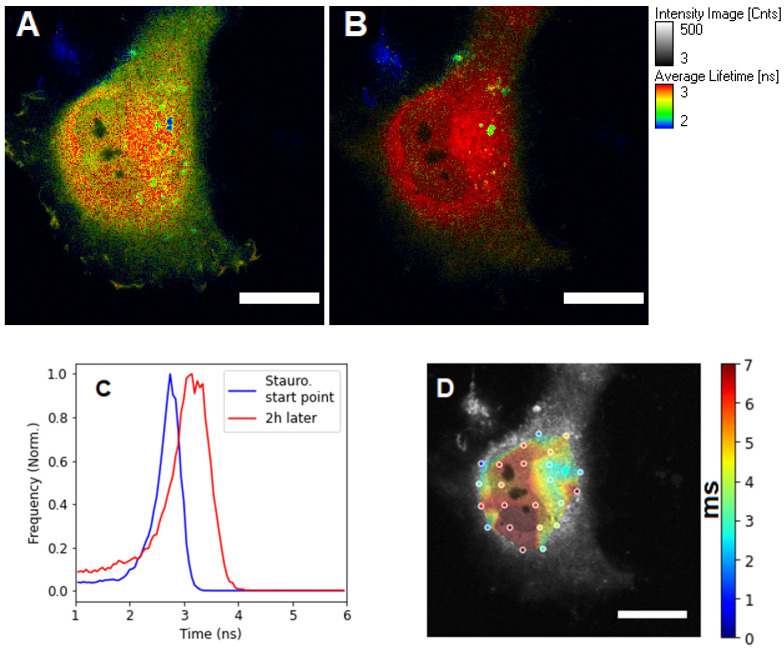
FLIM images of a HeLa cell transfected with a SAASoti-23-KFP sensor, (**A**) right after staurosporine and (**B**) after 2 h of incubation with staurosporine. (**C**) The distribution of the average fluorescence lifetimes is blue before and red after the induction of apoptosis for the whole frame (cell). (**D**) Diffusion time distribution image after 2 h of incubation with staurosporine. Images were acquired using a Microtime 200 confocal system (PicoQuant), excitation at 395/20 nm (red form generation), and 532 nm laser (red form excitation); emission was registered in the red channel. Scale bar = 20 μm.

Calibration is needed for the coefficients in Equation (1) and was performed on EGFP. Pinhole adjustment was carried out before measurements with the same EGFP solution (Table 1).

Then, the autocorrelation curves were processed sequentially according to the triplet model (Equation (1)) and preliminary fluorescence lifetime correlation spectroscopy (FLCS) processing. As follows from the curve in Figure 1, the photophysics data for the red form of the protein are well described by the triplet model. Since we use pulsed excitation of the red form with a 532 nm laser, and the red form arises as a result of wide-field irradiation with a 405 nm LED for 1 s and the subsequent collection of red photons for 5 s, we did not observe noticeable photobleaching, and the number of red form photons did not change over this period of time. For this type of measurement, the brightness of the red form is sufficient, as shown in Figure 1. This measurement method has been tested in in vitro experiments and also works well in live cells. The FLCS filter was obtained from time-correlated single photon counting (TCSPC) data which were recorded at the same time.

(1) $$G\left(\tau \right)=\left[1-T+Te^{\left(-\frac{\tau }{\tau_{T}}\right)}\right]\overset{n}{\underset{i=1}{\sum }}\rho_{i}{\left(1+\frac{\tau }{\tau_{i}}\right)}^{-1}{\left(1+\frac{\tau }{\tau_{i}\kappa^{2}}\right)}^{-\frac{1}{2}}\;\kappa =\frac{z_{0}}{w_{0}};\;V_{eff}=\pi^{\frac{3}{2}}w_{0}{}^{2}z_{0};\;\langle C\rangle =\frac{\langle N\rangle }{V_{eff}N_{A}};\;D_{i}=\frac{w_{0}{}^{2}}{4\tau_{i}};\;\;\overset{n}{\underset{i=1}{\sum }}\rho_{i}=\frac{1}{\langle N\rangle \left(1-T\right)}$$

where *V_eff_* (fl) is the effective detection volume; *z*_0_ (μm) is the effective axial focal size, 1/e^2^ of the intensity, calculated based on *V_eff_*; *w*_0_ (μm) is the effective lateral radius, 1/e^2^ of the intensity; *ρ_i_* is the *i* diffusion fraction; *τ_i_* (ms) is the diffusion time of the *I* component; *D_i_* (μm^2^/s) is the diffusion coefficient of the *i* component; 〈*N*〉 is the average number of molecules in the focal volume, calculated from *G* (0); 〈*C*〉 (nM) is the concentration of molecules in the focal volume; κ is the ratio of the axial to the lateral size; *T* is the fraction of molecules in the dark (triplet) state; and *τ_T_* (ms) is the lifetime of the dark state. After obtaining diffusion times for each point, color maps for cells were constructed with simple linear interpolation between points. An example of an autocorrelation curve for a triplet model is shown in Figure 1.

## 3. Results and Discussion

### Caspase-3 Activity after Induction of Apoptosis in Living Cells

First, HeLa cells were transfected with pcDNA3_V127T_SAASoti plasmid DNA containing no additional inserts (“free” SAASoti protein). As can be seen from Figure 2A, the protein diffuses freely throughout the cell, and from those presented in Figure 2B of the calculated diffusion times, heterogeneities in diffusion are weakly correlated with any of the cell compartments. 

The next step was to study the diffusion distribution of the FRET sensor for caspase-3, created based on the TagRFP-23-KFP sensor [17], but containing the SAASoti protein as a donor, denoted as the SAASoti-23-KFP sensor. Like its predecessor, the SAASoti-23-KFP sensor has an octameric structure consisting of four pairs of fusion proteins. Judging by the fluorescent image, this structure decreases penetration into the cell nucleus, so it looks dark (Figure 2C). It is known from the literature that constructs larger than 60 kDa and lacking localization signals have difficulty entering mammalian cell nuclei [26]. Otherwise, there is a uniform distribution of the sensor over the cytoplasm of the cell with rare events inside the nuclear pore. However, if we consider the time scale with retention times in the focal volume (Figure 2D,E), there is a sharp increase in the inhomogeneity of their values, and the regions with the greatest diffusion difficulties can be seen near the nucleus, a red area.

The most accurate control is the same cell before the introduction of staurosporine, which is an inducer of apoptosis. In mammalian cells, we observed the effect of FRET and an increase in the fluorescence lifetime after the induction of apoptosis in cells by the addition of staurosporine at a final concentration of 1 µM in the medium (Figure 2).

The response of cells to apoptosis inducers is individual and does not depend on the method of measuring enzymatic activity; this has been repeatedly described in the literature. [17,19]. This is why we analyzed only one individual cell. As it can be seen from Figure 2B, the average lifetime of the red form of the SAASoti protein upon activation of apoptosis in the cell varies from 2.7 to 3–3.5 ns, which indicates hydrolysis of the sensor construct by caspase-3 and impaired FRET.

Only the dynamics of the viscosity changes in relation to the starting value matter. This is why we analyze and compare only the initial measured parameters—diffusion times. The change in viscosity can be estimated by comparing the short retention times in the cytoplasm, and it must be taken into account that the cleavage of the substrate results in a partial change in its molecular weight. It was assumed that after hydrolysis of the sensor, a decrease in the diffusion time in the focal volume will be observed, i.e., an increase in the diffusion coefficient after the cleavage of the monomeric SAASoti protein from the octamer sensor design. However, if we compare the diagram distributions (Figure 2E) of diffusion times (~1/D) in cells expressing the SAASoti-23-KFP sensor before (blue) and after induction of apoptosis (gray), and cells expressing free SAASoti protein (orange), it can be noted that the diffusion of the cleaved sensor after the initiation of apoptosis is difficult, because diffusion times of SAASoti increase.

It is known that apoptosis is accompanied by a sharp increase in viscosity inside the cell [27]. As we were able to establish, this process is registered already during the activation of caspase-3, leveling the change in the size of the donor fluorescent protein due to its “release” from the FRET sensor.

Photochemical generation of the red form of SAASoti variants proceeds faster than that for the Dendra2 protein [21]. In the absence of specific signals of protein localization, the monomeric fluorescent protein SAASoti selected for further studies is localized in the cytoplasm of cells. Fast rates of green-to-red photoconversion are positive properties for the use of SAASoti for sub-diffraction microscopy and single molecule detection in living cells. The high intensity of the irradiated light (λ = 400 nm), which is required for most photoconvertible fluorescent proteins used in microscopy, can cause significant photochemical damage to living cells [28]. An advantage of SAASoti is that photoconversion requires less power of radiation toxic to cells.

**Figure 4:**
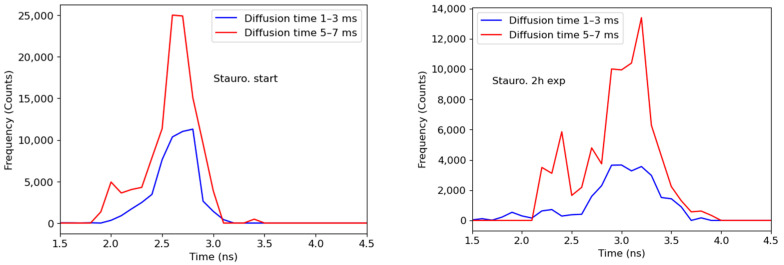
Distribution of average lifetimes with diffusion times in the range of 1–3 ms (6 points, blue) and 5–7 ms (8 points, red) in the vicinity of ±3 pixels of the FCS measurement points. Left—at the time of the addition of staurosporine; right—2 h after the addition of staurosporine.

The values clearly fall into two groups, as can be seen in Figure 2. We hypothesize that before apoptosis activation, the SAASoti-23-KFP substrate can partially bind to procaspase, which leads to the appearance of two retention times (Figure 2E). After the activation of apoptosis, we can observe both types of binding, with pro-caspase and active caspase, as follows from the lifetimes in the excited state for retention times of 5–7 ms in the nucleus and short 1–3 ms in the cytoplasm (Figure 4). Therefore, a total of four peaks are obtained (Figure 2E). This work is ongoing and the results of the proofs will be published separately.

## 4. Conclusions

The method we are developing belongs to the single cell category. This work demonstrates the possibility of using the photoconversion property of the SAASoti protein to obtain the required concentration range for the FCS method in a single cell. The SAASoti fluorescence lifetime for the red form is about 3.5 ns, which is higher than the average for fluorescent proteins (2.8 ns for eGFP) and makes it possible to observe changes in FRET in a wider dynamic range. The combination of FRET and FCS made it possible to establish that during the activation of caspase-3 during apoptosis, an increase in intracellular viscosity occurs. Thus, a fluorescence sensor, SAASoti-23-KFP, was obtained and successfully used, containing SAASoti as a fluorescence donor and allowing simultaneous study of enzymatic activity (caspase-3) and changes in viscosity by fluorescence correlation spectroscopy.

## Figures and Tables

**Table 1:** Calculated parameters for Equation (1).

κ (Fixed)	*ρ*	*τ_diff_* (ms)	*T*	*τ_T_* (ms)
5	0.044 ± 0.003	0.7 ± 0.2	0.4 ± 0.1	0.08 ± 0.05
